# Vibration Characterization of the Human Knee Joint in Audible Frequencies

**DOI:** 10.3390/s20154138

**Published:** 2020-07-25

**Authors:** Mohsen Safaei, Nicholas B. Bolus, Alper Erturk, Omer T. Inan

**Affiliations:** 1 School of Electrical and Computer Engineering, Georgia Institute of Technology, Atlanta, GA 30308, USA; inan@gatech.edu or; 2 Bioengineering Graduate Program, Georgia Institute of Technology, Atlanta, GA 30332, USA; nbolus@gatech.edu; 3 The George W. Woodruff School of Mechanical Engineering Georgia Institute of Technology, Atlanta, GA 30332, USA; alper.erturk@me.gatech.edu; 4 Wallace H. Coulter Department of Biomedical Engineering, Georgia Institute of Technology, Atlanta, GA 30332, USA

**Keywords:** vibration characterization, musculoskeletal injuries and disorders, diagnostic methods, modal analysis, knee joint

## Abstract

Injuries and disorders affecting the knee joint are very common in athletes and older individuals. Passive and active vibration methods, such as acoustic emissions and modal analysis, are extensively used in both industry and the medical field to diagnose structural faults and disorders. To maximize the diagnostic potential of such vibration methods for knee injuries and disorders, a better understanding of the vibroacoustic characteristics of the knee must be developed. In this study, the linearity and vibration transmissibility of the human knee were investigated based on measurements collected on healthy subjects. Different subjects exhibit a substantially different transmissibility behavior due to variances in subject-specific knee structures. Moreover, the vibration behaviors of various subjects’ knees at different leg positions were compared. Variation in sagittal-plane knee angle alters the transmissibility of the joint, while the overall shape of the transmissibility diagrams remains similar. The results demonstrate that an adjusted stimulation signal at frequencies higher than 3 kHz has the potential to be employed in diagnostic applications that are related to knee joint health. This work can pave the way for future studies aimed at employing acoustic emission and modal analysis approaches for knee health monitoring outside of clinical settings, such as for field-deployable diagnostics.

## 1. Introduction

The knee joint is, anatomically and biomechanically, one of the most complex joints in the human body [1]. Because of the role of knee in weight bearing (and particularly in cyclic motions such as walking), it is highly susceptible to injuries and disorders [2]. For instance, knee osteoarthritis is reported as the leading cause of activity limitation for adults in the United States [3]. Acute knee injuries are also very prevalent in young populations, especially athletes [4]. The late diagnosis of knee injuries and disorders can often lead to disability and/or the need for total knee arthroplasty [5]. Computed tomography (CT) and magnetic resonance imaging (MRI) are common techniques used to diagnose knee lesions. However, the former exposes the patient to radiation, and both imaging modalities are expensive and time consuming; moreover, neither approach could be employed outside of clinical settings, such as on the sports field or in rural settings.

The study of the sounds produced by a joint during articulation (often referred to as vibroarthrography, vibration arthrometry, or acoustic emission analysis) has been suggested by various researchers as a non-invasive diagnostic tool and an alternative to imaging techniques [6]. The emergence of modern instrumentation in the past four decades has made it possible for researchers to employ signal processing tools such as Fourier analysis, waveforms, spectra, and correlation functions to further characterize the joint sound and its correlation to different diseases and injuries [7]. Recent studies show that knee joint sounds can be used to distinguish between the healthy and pathologic joints in clinical [8] and ambulatory settings [9], by employing sensors, such as contact microphones and accelerometers, along with audio signal processing, feature extraction, and machine learning techniques.

While acoustic emission analysis has been shown to be a promising method of monitoring the health of the knee joint, the amount of information that can be extracted from the joint sounds is limited. Active vibration stimulation can be utilized in which the structure is excited externally, and the response is measured at the locations of interest in order to address this limitation. The study of the mechanical response of objects to an external stimulus can reveal information regarding material properties and vibratory characteristics [10]. Modal analysis techniques that are widely used in numerous industries [11] and various elastography methods used in the medical field are examples of the use of external stimuli to assess the mechanical properties of the system. Vibration-based characterization of the human body using external stimuli offers the ability to identify the modes of vibration, transmissibility, linearity, damping, and material properties of various tissues and anatomical structures, which could have a variety of diagnostic and therapeutic applications. For example, the response of the human body subjected to low frequency vibration and its therapeutic effects in athletes rehabilitating from injury is widely investigated in the existing literature [12]. The vibration characterization of the human skull to investigate the transmission of bone-conducted sound has been another attractive research area [13].

Numerous researchers have investigated the vibration transmission characteristics of the hand and arm due to the common use of rotary machines in industry. In 1976, Reynolds [14] investigated the vibration transmissibility of the human hand and arm subjected to a broadband vibration between 5 and 1000 Hz while using a handle attached to a shaker. Results showed that vibrations above 100 Hz are mostly isolated to the hand and fingers with small transmissibility to the arm. Moreover, it was observed that the vibration is even more localized to the areas directly in contact with the vibrating handle for frequencies between 150 and 200 Hz. The authors also concluded that the vibration attenuation is mostly due to soft tissue adjacent to the bone and not in the bone. Adewusi et al. [15] studied the transmission of handle vibrations to the wrist, elbow, and shoulder for bent arm and extended arm postures in six subjects. The results showed that an extended arm amplifies the vibration transmitted to the arm at low frequencies below 25 Hz and attenuates the vibrations above 25 Hz more when compared to a bent arm. Further, an increase in grip force was found to affect the vibration transmissibility at low frequencies, with smaller effects observed in high frequencies. Increased attenuation at higher frequencies (above 200 Hz) and the complexity of vibration measurement on human skin has limited the high frequency measurements on the human arm.

A study performed by Huang et al. [16] showed that coherence between excitation and response has the potential to be used to diagnose structural abnormalities in the hip. In another study by Conza et al. [17], the effect of pelvic ligaments on vibration characteristics of human pelvis was studied using fresh-frozen cadavers. It was shown that not all of the ligaments change the frequency response of the pelvis to external vibrations between 10 and 340 Hz. In a study performed by Soethoudt et al. [18], the in vivo dynamic characteristics of human pelvis were studied using an impact hammer and shaker tests on the anterior superior Iliac spine.

Despite the abundance of research performed on vibration behavior of various parts of the human body, there are few studies focused on vibration characteristics of the human knee joint. A modal analysis study of the human tibia has elucidated the effect of soft tissues and gradual transection on the modal characteristics. The human tibia experiences a rigid body vibration mode at around 165 Hz and a bending mode at around 300 Hz [19]. Impulse force transmission along the lower skeletal extremity revealed that a normal knee joint is able to attenuate 59% of the transient peak force applied to it by the tibia [20]. In a study that was performed by Dortmans et al. [21] in 1991, the nonlinear behavior of the knee joint was investigated in frequencies that were below 50 Hz. It was concluded that the knee joint is a nonlinear system that is based on the observable effects of the magnitude of the applied dynamic load on the frequency response of the system.

In this study, an investigation into the vibration characteristics of the human knee was conducted. The linearity and vibration transmissibility of the knee when stimulated at a distal location on the tibia were investigated. The transmissibility is defined as the ratio of the acceleration at the measurement point to the force at the excitation point (in the form of mechanical inertance, i.e., accelerance, which is the reciprocal of the apparent mass [22]. First, the accelerometer mounting method was examined in detail due to the importance of instrument placement. In addition, the effects of the excitation signal on the quality of measurement were explored, and proper excitation methods to perform linearity and transmissibility analyses were developed. The aim of this study is to obtain a better understanding of the vibratory properties of the knee and define the requirements for a robust vibration measurement tool. The results of this work will be used in future studies that employ these characterization techniques to diagnose and track various knee disorders and injuries.

## 2. Materials and Methods

A series of experimental tests were conducted in this study in order to investigate the vibration characteristics of the human knee joint. Mechanical stimulation of the knee joint on the tibia and response measurement on the femur using two accelerometers were utilized to obtain information regarding the linearity and vibration transmissibility of the joint. It has been shown that the accelerometer placement on an object can considerably affect the outcomes of a vibration measurement [22]. Therefore, the effects of various adhesive materials on measurement performance of accelerometers were initially investigated in order to find the best mounting method. The selected adhesion method was then used in the knee measurement studies to place the accelerometers on each subject’s leg. The study was approved by the Georgia Institute of Technology Institutional Review Board and all of the subjects gave their informed consent before they participated in the study. Six healthy subjects (five male, one female, 25.1 ± 7 years, 74.3 ± 22.5 kg, 178.8 ± 18.5 cm) with no history of major knee injury were enrolled, and all of the measurements were conducted on the right leg.

### 2.1. Selection of Tape for Accelerometer Mounting

**Figure 1:**
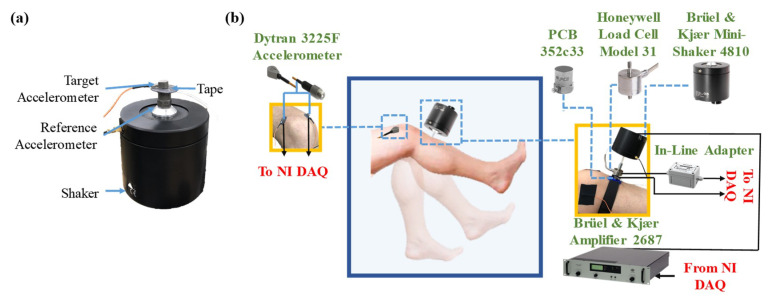
An overview of (**a**) accelerometer mounting test setup on the electrodynamic shaker and (**b**) human joint vibration measurement setup, including a modal shaker and amplifier, a load cell and a reference accelerometer attached to the shaker, and a pair of measurement accelerometers placed medial and lateral to the proximal end of the patella; the vibration transmission through the joint is measured at three different flexion angles when the joint is stimulated by the shaker at a tibial location.

Three different double-sided tape options used to place the accelerometer on the skin were considered, including Rycote Lavalier Stickies double-sided pads (Rycote Microphone Windshields Ltd., Gloucestershire, UK), 3M medical tape 1509 (3M, St. Paul, MN, USA), and Elizabeth Transparent Double-Sided Tape (Elizabeth Craft Designs, Inc., Evergreen, CO, USA). The Rycote, 3M, and Elizabeth tapes have a thickness of 2 mm, 80 micron, and 70 micron, respectively. Note that the Elizabeth tapes are not medical grade product used due to availability, however, according to company the tape material is not hazardous. For future use, equivalent medical grade tapes with the same thickness will be utilized. In addition, Cyanoacrylate glue (super glue) was used as the reference mounting method. A test setup consisting of a B&K 4810 shaker (Brüel & Kjær, Nærum, Denmark), a reference accelerometer (PCB 352c33, PCB Piezotronics Inc., Drew, NY, USA), and a target accelerometer (Dytran 3225F, Dytran Instruments Inc., Chatsworth, CA, USA) were utilized (Figure 1a) in order to investigate the performance of each tape.

The same equipment was later used in vibration measurements on the human knee. The target accelerometer was placed on the top of the reference accelerometer using double-sided tape, and the reference accelerometer was attached to the shaker table while using a mounting screw. A swept sine signal with a duration of 20 s and frequency ranging from 50 Hz to 10 kHz was used to excite the shaker table. The accelerometers were connected to a USB 4431 data acquisition module with IEPE-activated analog channels (National Instruments, Austin, TX, USA) in order to measure the acceleration at a sampling rate of 50 kHz. Upon measuring acceleration with each double-sided tape option, the target accelerometer was separated from the reference accelerometer and replaced to investigate the effect of tape degradation when reused. The measured accelerations were filtered using a Kaiser-window finite impulse response bandpass filter with frequency bandwidth equal to that of the excitation signal. The Kaiser filter is used due to its ability of stopband attenuation and the independency of the bandpass and stopband ripple size from window length [23]. The frequency response of the target accelerometer with respect to the reference accelerometer was calculated in MATLAB (Mathworks, Natick, MA, USA) at 8192 frequency points while using the transfer function estimator $H_{1}$ as stated in Equation (1) [24].

(1) $$H_{1}\left(f\right)=\frac{S_{xy}\left(f\right)}{S_{xx}\left(f\right)}$$

where, $S_{xy}\left(f\right)$ is the cross power spectral density of input (x) and output (y), and $S_{xx}\left(f\right)$ is spectral power density of input. Using estimator $H_{1}$, the random noise in the output can be minimized during the averaging in the cross spectrum [25].

### 2.2. Linearity of the Human Knee Joint

Using a vibration setup, as shown in Figure 1b, the linearity (homogeneity) and vibration transmissibility of the knee joint in six subjects was studied. The setup consisted of an excitation unit including a 4810 mini-shaker, a load cell model 31 (Honeywell International Inc., Charlotte, NC, USA), and a PZB 352c33 accelerometer attached to the tibia next to the tuberosity. The shaker was pressed against the tibia with a compression load of around 5 N that was measured by the load cell to ensure that the subject’s leg is under compressive force for the entire time of measurement. A 10 N maximum force was considered as the comfortable compression level based on an initial feedback from subjects. The maximum input acceleration over the course of measurement was 5 m/s^2^, which is far below the safe threshold defined by International Standard Organization (ISO 5349-1:2011) for exposure to vibration for less than 5 min. [26]. The transverse movement of the shaker head relative to the skin was constrained using a three-dimensional (3D) printed component, fixed on the tibia with an elastic band, minimizing the effects of lateral motion [18]. Two Dytran 3225F accelerometers were placed medial and lateral to the patella proximal to the joint using Elizabeth tape selected based on the analysis performed in the previous section.

**Figure 2:**
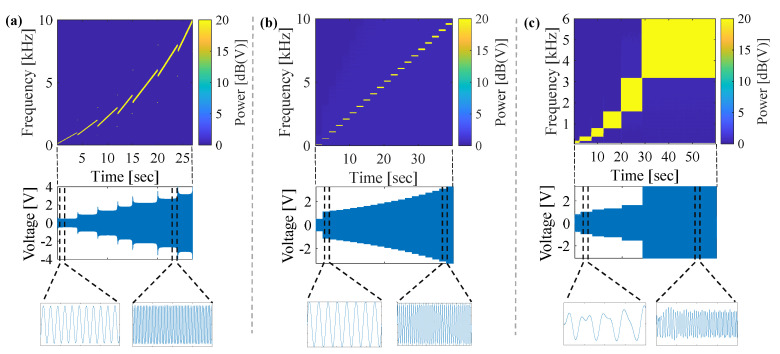
Two different excitation signals designed for linearity analysis: (**a**) a high-resolution subsequential multisine signal with a resolution of 0.5 Hz, (**b**) a stepped-sine signal with 500-Hz increments, and (**c**) a customized subsequential, equidistance, quasi-logarithmic multisine designed for vibration transmissibility tests.

Two excitation signals were used to achieve a coherent measurement between the input (load cell) and output (Dytran accelerometers). Initially, a variable amplitude subsequential multisine excitation with high frequency resolution of 0.5 Hz was used to stimulate the joint. Due to low input-output coherence of the measurements, subsequently, a stepped-sine signal was used to measure the linearity of knee joint. Figure 2b shows the stepped-sine excitation signal with frequency increments of 500 Hz, lowest frequency of 100 Hz, highest frequency of 9600 Hz, and individual sines of 2 sec. The stepped-sine excitation provides us with higher spectral power into each excitation frequency and sufficient number of averages to reduce the random measurement noise and, as a result, higher signal-to-noise ratio (SNR) in measured signals. Input-output coherence and SNR were used to evaluate the quality of measurements using different excitation signals. SNR was calculated by taking the ratio of the fundamental frequency amplitude to the RMS amplitude of the noise.

The amplitude of the stepped-sine excitation signal was multiplied by 1.2, 1.4, 1.6, 1.8, and 2, the equivalent effect of which is an increase of 1.6, 3, 4, 5.2, and 6 dB, respectively, in order to investigate the linearity of knee joint under different excitation levels. The difference between the frequency response results obtained from each accelerometer was calculated using logarithmic spectral distance (LSD) using Equation (2) [27]

(2) $$LSD=\sqrt{\frac{1}{N}\sum {\left(20\log_{10}\frac{\left|H_{12}\left(f\right)\right|}{\left|H_{11}\left(f\right)\right|}\right)}^{2}}$$

where $H_{11}\left(f\right)$ and $H_{12}\left(f\right)$ are the frequency responses of first and second measurements, respectively, at frequency f, and N is the total number of frequency points used in the frequency response measurement. The total number of frequency points was equal to the number of sine signals used in the linearity analysis (20 sine signals).

### 2.3. Vibration Transmissibility of Human Knee Joint

Initial results that were obtained from joint linearity studies using a high frequency resolution signal and stepped-sine showed that the frequency resolution and length of the excitation signal need to be carefully selected to achieve a coherent and reliable measurement. While stepped sine can easily be used to identify the transfer function of, and the nonlinearities in, a structure, the measurement time can be very long. In contrast, a well-designed multisine with a low crest factor can be used to excite multiple frequencies of the system simultaneously and, therefore, reduce the measurement time. However, in systems with low SNR, the measurement time to average out the random noise for multisine can be long [28]. Accordingly, a customized subsequential, equidistant quasi-logarithmic multisine excitation signal was designed and used in vibration transmissibility tests. The spectrogram of the designed signal, together with the time domain representation, is shown in Figure 2c. The signal excites frequencies from 100 Hz to 6400 Hz in six multisines of different amplitudes. The amplitude was increased for higher frequencies due to decreasing excitation amplitude of the shaker at high frequencies and reduced SNR in response. The frequency range, amplitude, duration of each sub-signal, number of available frequencies, frequency resolution, and number of repetitions of each sub-signal are listed in Table 1 for reference. Frequency ranges were selected in such a manner that the super harmonics of the fundamental frequency were not excited, and the range and frequency resolution were logarithmically increased. The number of signal repetitions were increased at higher frequencies to provide sufficient numbers of averaging and to increase the measurement SNR. The repeatability of measurements was investigated by repeating the tests three times on each subject. To investigate the variation of vibration transmission within subjects, the frequency response diagrams obtained from six subjects were compared, and the deviations from an average diagram were calculated. Additionally, the leg posture was varied by conducting experiments at the three sagittal-plane knee angles of 10°, 40°, and 70° to study the effects of knee position on vibration transmissibility. LSD parameter was used in all studies to measure the differences between various transmissibility diagrams.

## 3. Results

### 3.1. Selection of Tape for Accelerometer Mounting

**Figure 3:**
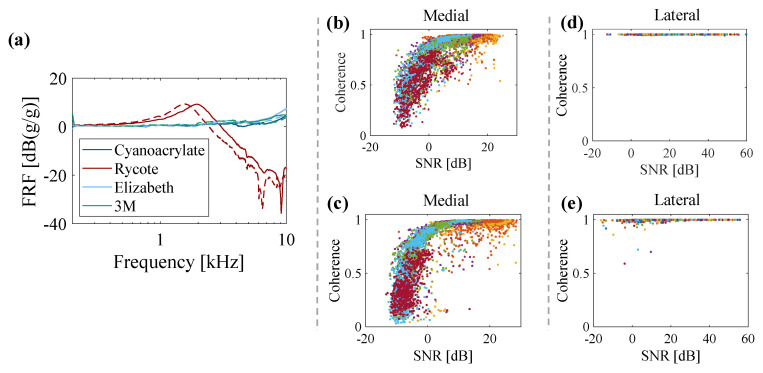
(**a**) Frequency response function (FRF) of target accelerometer using various mounting methods (solid lines: initial use, dashed lines: reuse); response SNR and coherence measurement for high frequency resolution signal for (**b**) medial accelerometer and (**c**) lateral accelerometer; response SNR and coherence measurement for stepped-sine signal for (**d**) medial accelerometer and (**e**) lateral accelerometer.

Initially, three types of tapes were used to place an accelerometer on the shaker table and evaluate the performance of each mounting method. The frequency response results for seven mounting cases are presented in Figure 3a, where solid lines indicate the initial use cases and dashed lines indicate the reuse cases. The results suggest that the thin tapes behave in a similar manner to the Cyanoacrylate glue upon both initial use and reuse. In contrast, the Rycote double-sided tape frequency response deviates from the reference response at both low and high frequencies. In particular, a decrease of 20 dB in the frequency response at frequencies higher than 8 kHz is noticeable. This adhesive material was selected as the accelerometer mounting technique for the remainder of this study, given that the frequency response better preserves high frequency information, and the low cost and availability of the Elizabeth tape.

### 3.2. Linearity of the Human knee Joint

#### 3.2.1. Selection of excitation signal

Due to the complexity and high damping of the human knee joint, the quality of acceleration measurements as a result of mechanical stimulation is highly dependent on the excitation vibration signal. The input-output coherence of the measurements as well as the SNR for the two different excitation signals (multisine vs stepped-sine) are plotted in Figure 3b–e. The coherence and SNR values are calculated for all of the subjects at different frequency ranges, as shown in Figure 2a,b for each sub-signal. The high frequency resolution multisine is not able to provide a quality measurement due to low SNR in response and, therefore, low coherence. High damping characteristics of the human knee joint [20] along with the effects of adjacent soft tissues [17] (particularly at high frequencies) are the key parameters affecting the quality of measurement. In contrast, the stepped-sine signal excites the knee joint with sufficient power and length that the response measurements yield a very good coherence, even when the SNR is relatively small. Therefore, the stepped-sine excitation was selected as the input for the linearity study.

#### 3.2.2. Linearity Analysis

**Figure 4:**
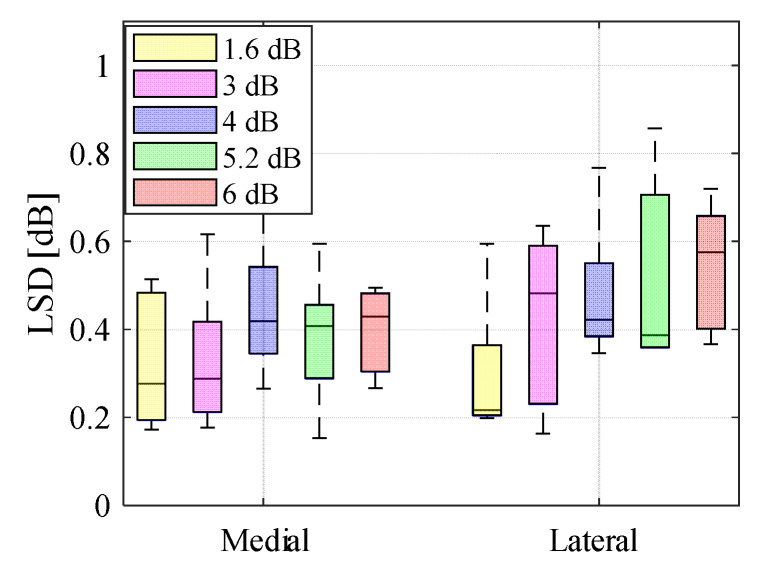
Box plot of log-spectral-distance calculated for five different input excitation levels of 1.6, 3, 4, 5.2, and 6 dB with respect to an initial excitation level of 1 V.

After stimulating the knee joint with the stepped-sine excitations of various amplitudes, the frequency response was calculated for both accelerometers. For a linear system, the frequency response of the system when the amplitude is changed remains unperturbed in shape, but simply increases or decreases in magnitude [29]. The LSD parameter, as defined in Equation (2), was used to measure the deviation of response from an initial signal for each signal level. Figure 4 shows a box plot of the LSD values calculated from the frequency response measurements at 5 different stimulation levels with respect to the initial amplitude from 6 subjects. The median deviation of response for all the amplitudes is less than 0.6 dB (1.07 times in linear scale) relative to an initial excitation level. Very small changes in frequency response and inconsistent variations in the quantities of LSD values suggest that the human knee joint can be treated as a linear system in the frequency range of 100 Hz to 10 kHz and input forces below 5 N.

### 3.3. Vibration Transmissibility of the Human Knee Joint

#### 3.3.1. Repeatability of Vibration Transmission Measurements

**Figure 5:**
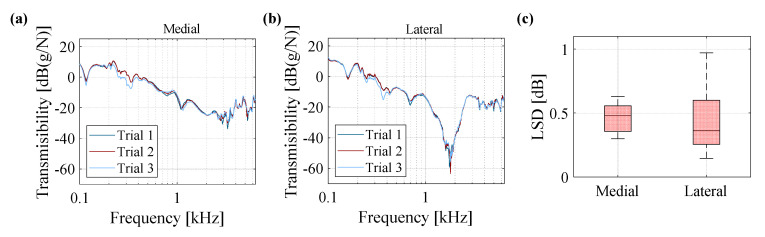
Vibration transmission of knee joint presented for one subject and for three trials to measure the repeatability of tests for (**a**) medial accelerometer and (**b**) lateral accelerometer; and, (**c**) box plot of logarithmic spectral distance (LSD) values calculated for six subjects at medial and lateral accelerometers.

The vibration transmission in the human knee joint was investigated using a customized stimulation signal illustrated in Figure 2c. The measurements were repeated for each subject three times by detaching and reattaching the stimulation and sensor components to evaluate the repeatability of the measurement. The transmissibility (frequency response) of one subject at a knee angle of 40° is presented in Figure 5a,b for the accelerometers placed on the medial and lateral sides of the patella on the femur. The transmissibility diagrams for the three tests have inappreciable deviation from each other. The maximum LSD values with respect to the average transmissibility of the three trials were calculated, and a box plot of these values is shown in Figure 5c for medial and lateral accelerometers, to quantify the deviation values for all six subjects. The LSD calculations yield a median value of less than 0.5 dB (1.06 times in linear scale) across subjects, which supports the repeatability of the tests. Nevertheless, note that the time between different repeatability measurements of each subject has been less than five minutes.

#### 3.3.2. Inter-Subject Variation of Vibration Transmissibility

**Figure 6:**
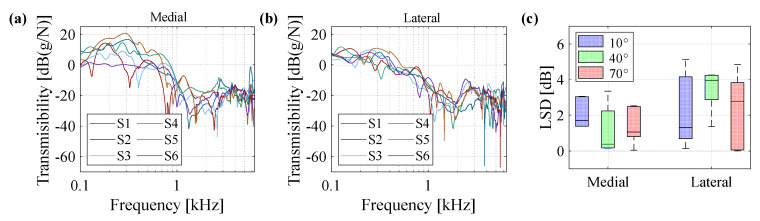
Vibration transmission of knee joint obtained from six subjects and for the knee angle of 40° for (**a**) medial accelerometer and (**b**) lateral accelerometer; (**c**) box plot of LSD values calculated for six subjects at knee angles of 10°, 40°, and 70°, for medial and lateral accelerometers.

Figure 6a,b show the transmissibility plots of all subjects measured at a knee angle of 40° from medial and lateral accelerometers, respectively. The results demonstrate that while all of the subjects present a similar overall behavior, there are substantial differences in the location (frequency) and amplitude of resonances and anti-resonances observed in the frequency response as well as overall transmissibility level of each subject. The differences in transmissibility graphs compared to the average diagram of all subjects is calculated in terms of LSD and plotted in Figure 6c for three knee angles of 10°, 40°, and 70°, and for the two accelerometers. The box plot representation of results suggests considerably higher differences in the measured responses between subjects as compared to the repeatability test results at all knee angles.

#### 3.3.3. Effects of Knee Angle on Vibration Transmission

**Figure 7:**
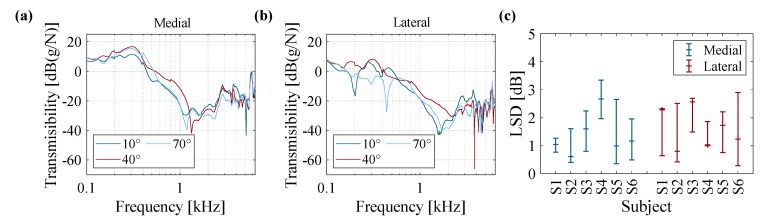
Vibration transmission of knee joint obtained from one subject at different knee angles of 10°, 40°, and 70° for (**a**) medial accelerometer and (**b**) lateral accelerometer; (**c**) a plot of median, maximum and minimum LSD values calculated for six subjects at knee angles of 10°, 40°, and 70° for medial and lateral accelerometers.

The knee angle was varied between 10°, 40°, and 70° to study the effect of knee flexion on the vibration transmission of the joint. The results for one subject obtained from medial and lateral accelerometers are plotted in Figure 7a,b, respectively: changing the angle can lead to a small variation in transmissibility of the knee. The LSD values that represent the deviation of transmissibility data of the three knee angles from the average quantities are plotted for the six subjects in Figure 7c. Overall, the knee angle is identified as a major contributor affecting the vibration behavior of the joint, which needs to be carefully adjusted between tests in comparative analyses of knee. Conversely, the variation of transmissibility of knee as a function of angle is lower than the inter-subject variations, and the overall trend in the graphs suggests less variations when the angle is varied.

#### 3.3.4. Vibration Transmissibility from 100 Hz to 10 kHz

**Figure 8:**
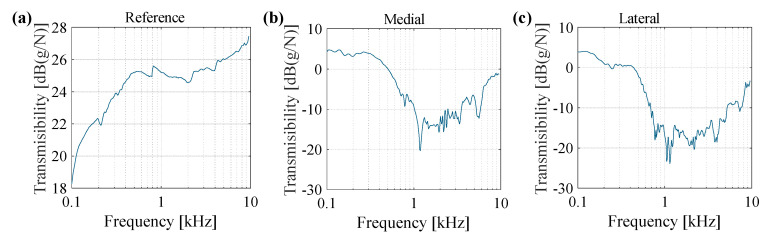
Vibration transmission of knee joint obtained from a representative subject at knee angle = 40°, (**a**) reference accelerometer, (**b**) medial accelerometer, and (**c**) lateral accelerometer.

In the previous sections, the vibration transmissibility of the knee joint was measured for the frequency range of 100 Hz to 6400 Hz. Stimulating the knee joint at frequencies higher than 6400 Hz requires long measurement times due to low SNR of the response signals and the need for a large number of repetitions to remove the random noise. Long-duration measurements make the subjects uncomfortable, as they cannot hold their leg in a still position throughout the measurement period, which results in poor input-output coherence as well as reduced repeatability. However, using a fast multisine similar to the signals that are presented in Figure 2a, the overall vibratory behavior of the knee can be assessed. Figure 8 shows the vibration transmissibility obtained for one subject calculated from the three accelerometers. The overall shape of the transmissibility graphs for all subjects is similar, thus the results are presented for a representative subject. Reference accelerometer was placed on the tip of exciter and measured the driving point acceleration. The driving point inertance frequency response, which is inversely proportional to the apparent mass, is shown in Figure 8a. It is evident in the graph that the frequency response increases at low frequencies (in the range of hundreds of Hz), where the apparent mass of the knee decreases with increasing frequency. The transmissibility graphs in Figure 8b,c for measurements at the medial and lateral accelerometers present a reduction in the frequency response at frequencies below 1 kHz and an increase in the transmissibility at frequencies above 3–4 kHz.

## 4. Discussion and Conclusion

This paper presents a fundamental study describing the vibration characteristics of the human knee joint. Using an external vibration stimulation, the linearity and vibration transmissibility of the knee were investigated for six subjects. It is demonstrated that the knee can be treated as a linear system at frequencies between 50 Hz and 10 kHz under a compression force of less than 5 N. In addition, each subject’s knee presents unique variations in its vibration signature that are substantially different from that of other subjects. The inter-subject transmissibility differences are expected given variations in the structure of knee joint in different subjects due to bone density [30], muscle-tendon forces [31], articulating surfaces and contact mechanisms [32], dimensions and mechanical properties of organs [33], and skin [34]. However, the overall decreasing trend of transmissibility at higher frequencies observed in all subjects presents a consistent behavior amongst subjects that can be potentially used in the future when vibration stimulation is used as a diagnostic tool.

Leg posture was also found to be an important factor in vibration analysis of the knee, as it introduces noteworthy changes in the transmissibility of the joint as well as a slight effect on the overall shape of the system’s frequency response. Because the knee joint and surrounding tissues and fluids form a complex structure comprised of various active and passive components, such as muscles, tendons, articular cartilages, meniscus, bone, fat, skin, synovial membrane, etc., various degrees of flexion can change the vibration characteristics of the joint [35]. In particular, the transmissibility at the lateral location demonstrated a more noticeable change. This can be attributed to more appreciable changes in the contact pattern on the lateral plateau of knee as a result of flexion as well as relatively greater distance between the lateral accelerometer and excitation location as compared to the medial accelerometer [36].

Perhaps the most compelling result from this paper, and one that lays the foundation for future research in the area of advancing the understanding of acoustic and vibration propagation through the knee, is the quantification of vibration transmissibility through the joint. It is reported in the existing literature that combined rigid body motion and modal vibration can be observed in the human leg at low frequencies [37]. Given the relatively high apparent mass (low inertance), it can be concluded that the subject’s knee is experiencing such motions for frequencies below 1 kHz (specifically, a careful investigation of Figure 8a reveals the presence of a mode around 400–500 Hz, as confirmed with the phase graph not shown here). Above this low frequency range (> 1 kHz), any potential modes of knee origin are not easily detectable while using the driving point measurement due to high damping characteristics of the joint [14], resulting in small reflections of traveling or standing waves. The measurements of the other two points (medial and lateral accelerometers) are better suited to explore the effect of joint dynamics at frequencies higher than 1 kHz. The subsequent decrease in the inertance frequency response after this mode (in its mass controlled region, i.e., from around 500 Hz to 1 kHz) is expected [22,38]. The presence of rigid body modes of vibration at frequencies lower than 1 kHz suggests that more information regarding the internal structure of joint can be acquired at frequencies beyond 1 kHz.

Several resonances and antiresonances are observed in the high frequency range (>1 kHz) that suggest the presence of highly complex dynamics with many degrees of freedom. When considering the complexity of the knee joint, including solid-solid interfaces, solid-fluid interfaces, and a high number of active and passive tissues, the occurrence of such internal resonances is not surprising. In both the medial and lateral accelerometer measurements, a substantial attenuation of the transmitted vibration energy is observed in this frequency range (for a few kHz above 1 kHz). This same frequency range (of reduced transmission through the leg) is expected to be the frequency range in which the complex dynamics and dissipative characteristics of the knee joint are manifested. Previous studies on the knee joint acoustic emissions also suggest that the majority of spectral power is available at frequencies below 1 kHz [8], which can be due to the attenuation that is observed at higher frequencies (<1 kHz) in this study. Another observation is a gradually increasing trend of the frequency response for frequencies above 3–4 kHz. Given the higher spatial sensitivity of elastic waves at higher frequencies as a result of shorter wavelength, an increase in transmissibility shows the potential of high frequency component stimulations in detecting the internal defects generated by injury or disease inside the knee joint.

This study has some limitations that should be noted. First, the participants were all healthy, young subjects with no known knee injuries or disorders. The vibratory properties of the knee depend on the musculoskeletal condition, which is highly correlated to the age of the subjects due to cell senescence, aging in the cartilage matrix, and oxidative stress and damage [39]. Future work will be needed to include specific age groups at a similar musculoskeletal development stage and examine how acute knee injuries and/or disorders affecting the internal structures may impact the transmissibility and vibration properties of the joint. Additionally, the number of subjects studied was relatively small, with only six subjects being involved in the testing. Importantly, more female subjects need to be included in the study to accommodate the effects of gender-related musculoskeletal variations. This is not a major limitation at this stage since this work primarily establishes a methodology that can be used in future studies to examine in more depth the differences across subjects in the phenomena observed in this work. A quantitative analysis of vibroacoustic properties of the knee in various age and gender populations can be conducted in the future using the methodology described herein for a larger number of subjects. Finally, the transducer used for exciting the knee was limited in the power that it could deliver and, thus, the higher frequency assessments were limited to <6.4 kHz. In future work, vibration exciters capable of providing sufficient power at frequencies higher than 6.4 kHz will be utilized along with non-contact sensors (e.g., laser-doppler vibrometer) to reveal more information regarding knee joint vibroacoustic behavior.

Future work will also involve estimating a subject-specific transmissibility diagram of the knee joint with long intervals between measurements and after physical activities. Overall, the insights gained regarding the vibratory properties of the knee will be used in future studies to employ mechanical stimulation for identifying various internal disorders and injuries present in the knee joint. Using the developed experimental procedure in this study, vibration analysis will be conducted in healthy and defected cadaver and animal models to investigate the ability of vibration stimulation to diagnose various types of injuries and disease. Identifying the sensitive frequency ranges and features of transmissibility diagrams to specific knee disorders, mechanical stimulation has the potential to be used outside of clinical settings as a wearable technology.

## Figures and Tables

**Table 1:** List of sub-signals used to form a customized multisine signal for stimulation of knee joint.

Signal Number	Frequency Range (Hz)	Amplitude (V)	Duration (ms)	Number of Frequencies	Frequency Resolution (Hz)	Number of Repetitions
1	100–200	0.8	500	51	2	5
2	200–400	1.0	250	51	4	20
3	400–800	1.2	125	51	8	40
4	800–1600	1.3	62.5	51	16	120
5	1600–3200	1.6	31.25	51	32	280
6	3200–6400	3.3	15.625	51	64	2000
